# Measures of upper limb function for people with neck pain: a systematic review of measurement and practical properties (protocol)

**DOI:** 10.1186/s13643-015-0034-2

**Published:** 2015-04-07

**Authors:** Ahmad Salah Eldin Alreni, Deborah Harrop, Anil Gumber, Sionnadh McLean

**Affiliations:** Faculty of Health and Wellbeing, Sheffield Hallam University, Montgomery House, 32 Collegiate Crescent, Collegiate Campus, Sheffield, S10 2BP UK; Faculty of Health and Wellbeing, Sheffield Hallam University, Mercury House, 36 Collegiate Crescent, Collegiate Campus, Sheffield, S10 2BP UK

**Keywords:** Upper limb function, Disability, Neck pain, Outcome measures, Quality, Psychometric properties

## Abstract

**Background:**

Upper limb disability is a common musculoskeletal condition frequently associated with neck pain. Recent literature has reported the need to utilise validated upper limb outcome measures in the assessment and management of patients with neck pain. However, there is a lack of clear guidance about the suitability of available measures, which may impede utilisation. This review will identify all available measures of upper limb function developed for use in neck pain patients and evaluate their measurement and practical properties in order to identify those measures that are most appropriate for use in clinical practice and research.

**Methods/design:**

This review will be performed in two phases. Phase one will identify all measures used to assess upper limb function for patients with neck pain. Phase two will identify all available studies of the measurement and practical properties of identified instrument. The COnsensus-based Standards for selection of health Measurement INstrument (COSMIN) will be used to evaluate the methodological quality of the included studies. To ensure methodological rigour, the findings of this review will be reported in accordance with the Preferred Reporting Items for Systematic Review and Meta-Analysis (PRISMA) guideline.

**Discussion:**

Optimal management of patients with neck pain should incorporate upper limb rehabilitation. The findings of this study will assist clinicians who seek to utilise suitable and accurate measures to assess upper limb function for a patient with neck pain. In addition, the findings of this study may suggest new research directions to support the development of upper limb outcome measures for patients with neck pain.

**Systematic review registration:**

PROSPERO CRD42015016624

## Background

Upper limb dysfunction is a common musculoskeletal condition [1]. The prevalence of upper limb dysfunction at any given point of time has been estimated as 20% to 53% in the working population of Western industrial countries. The lifetime prevalence of upper limb dysfunction is greater than 70% [2,3]. Upper limb dysfunction can arise from a spectrum of clinical conditions including neck pain [4,5]. This can have a substantial effect on quality of life, work absenteeism, and loss of productive capacity and is therefore a substantial socioeconomic burden for patients and society [6].

It is not clear what proportion of neck pain sufferers in the general population experience associated upper limb disability, but among patients with neck pain, upper limb function is often impaired [7-11]. An extreme example of this is cervical radiculopathy, where neurological pathology may lead to pain, motor weakness, sensory deficit, and loss of function in the neck, shoulder, upper arm, or forearm [12,13]. However, non-specific neck pain has also been shown to have a substantial impact on upper limb function. In one Australian study of patients with non-specific neck pain (*n* = 103), 80% reported upper limb functional limitation [11].

In their prospective cohort study, McLean *et al*. [5] investigated the association between non-specific neck pain severity and upper limb disability and demonstrated that there was a significant and substantial positive correlation between the two (*r* = 0.799, *P* < 0.001, *n* = 151). These studies indicate that the impact of neck pain on upper limb function can be substantial and severe.

Clinicians should carefully assess upper limb functional capacity during the examination of patients with neck pain and, where indicated, incorporate upper limb rehabilitation in their management [5,11]. This suggests the requirement of valid and reliable measures to assess and monitor upper limb disability during the management process [4]. However, there is currently a lack of clear guidance about the suitability of available outcome instruments for clinical practice and research [14]. This review will identify all available measures of upper limb function developed for use in neck pain patients and evaluate their measurement and practical properties in order to identify those measures that are most suitable for use in clinical practice and research practice. This will include all types of outcome measures such as patient-reported outcomes (PROs), clinician-reported outcomes (ClinRos), observer-reported outcome (ObsRO), and performance-based outcome measures (PreFO).

## Methods/design

The proposed review will be conducted in two phases. Phase one will identify all measures used to assess upper limb function for patients with neck pain. Phase two will identify published and unpublished studies of the measurement and practical properties of the identified measures. The methodological quality of the developmental and evaluative studies will be assessed against the COnsensus-based Standards for selection of health Measurement INstrument (COSMIN) guidelines [15,16]. To ensure methodological rigour, the results of this review will be reported following the Preferred Reporting Items for Systematic Review and Meta-Analysis (PRISMA) guideline [17].

### Phase one - identification of measures

The following databases will be searched from their inception: Allied and Complementary Medicine Database (AMED) (OvidSP), CINAHL Complete (EBSCO), the Cochrane Library (Wiley), MEDLINE (EBSCO), PubMed (US National Library of Medicine), PsycINFO (ProQuest), SPORTDiscus (EBSCO), Web of Science (Thomson Reuters).

### Search strategy

A search strategy combining title/abstract words and, where available, database-controlled vocabulary terms relating to upper limb function and neck pain will be used to locate all measures used to assess upper limb function in neck pain patients.

The search strategy is detailed below. Explanation of the syntax used:/= MeSH; * = denotes any character/s; *n* =adjacency within x words; “” = phrase search.

(“upper limb”/“upper extremity” n5 function*, OR “upper limb”/“upper extremity” n5 dysfunction*, OR “upper limb”/“upper extremity” n5 abilit*, OR “upper limb”/“upper extremity” n5 disabilit*, OR “upper limb”/“upper extremity” n5 capacit*, OR “upper limb”/“upper extremity” n5 disorder*, OR “upper limb”/“upper extremity” n5 problem*, OR “upper limb”/“upper extremity” n5 pain*, OR “upper limb”/“upper extremity” n5 deficit).

AND

(“neck”/“cervical spine” n5 function*, OR “neck”/“cervical spine” n5 dysfunction*, OR “neck”/“cervical spine” n5 abilit*, OR “neck”/“cervical spine” n5 disabilit*, OR “neck”/“cervical spine” n5 problem*, OR “neck”/“cervical spine” n5 pain*, “neck”/“cervical spine” n5 disc*, “neck”/“cervical spine” n5 degenerative, OR “neck”/“cervical spine” n5 diseas*, OR “neck”/“cervical spine” n5 disorder*, OR “neck”/“cervical spine” n5 deficit).

A RefWorks database will be used to manage all references. Two reviewers will independently screen the titles and abstracts of the studies retrieved from the literature search. In the case of disagreement between the two reviewers, a third reviewer will be used to make the decision regarding inclusion of the study. Any study will be considered for inclusion without restriction of design or publication date if 1) they involve adults, age ≥ 18, with neck pain, which is defined here as a dysfunction of the cervical structure and 2) at least one of the measures aimed to measure upper limb disability, which is defined here as any difficulties or limitations an individual may have in executing upper limb activity [18]. Studies will be excluded if they 1) are not available in English, 2) involve participants under 18 years of age, and/or 3) involve participants with a disorder other than neck pain. Following title and abstract screening, selected full-text articles and reference lists of the studies retrieved by the literature search will be reviewed for inclusion. Clearly defined and reproducible outcome measures of upper limb function in the context of neck pain in selected studies will then be identified and collated.

### Phase two - identification of the development and/or evaluative studies

The search in this phase will aim to identify studies related to the development or evaluation of measures identified in phase one. The databases identified in phase one will be used to perform further specific searches for each of the identified measures. If required, a sensitive search filter [19] for locating studies on measurement properties of measurement instruments will be applied to any searches producing large result sets. To ensure comprehensiveness, key authors and experts will be identified and contacted for additional relevant published or unpublished studies to include in this review. Studies will be included if they are available in English and their aim was to develop an instrument to measure upper limb function in patients with neck pain or to evaluate one or more of the practical properties of an instrument. Two independent reviewers will screen all titles, abstracts, and full-text studies retrieved from the literature search. A third reviewer will resolve any disagreement between first two reviewers’ inclusion/exclusion of studies. Finally, reference lists of selected full-text articles will be screened to identify additional relevant studies.

### Data extraction and study evaluation

Selected studies in this review will be evaluated in accordance with the modified COSMIN checklist [15,16], and data will be extracted to a standardised form, which has been used in other similar studies [20-22]. This will ensure the collection of data required to evaluate the quality of identified outcome measures and the methodological quality of included studies.

Data extraction will capture information regarding 1) study sample (age, gender, diseases (neck pain and upper limb functional limitation), intervention, setting, country, and recruitment methods), 2) instrument general characteristics (construct, subscale, items, version, version language, and tasks (performance-based)), and 3) instrument properties, which will seek evidence of the following measurement and practical properties: reliability (internal consistency (unidimensionality of the scale and Cronbach’s alpha), measurement errors (smallest detectable changes (SDC), minimal important change (MIC)), validity (content, construct, criterion), responsiveness (content, criterion-approach, and construct-approach), precision (measurement floor and ceiling effect), acceptability (measurement completion rates, missing value, completion time, comprehension level, and special requirement), and feasibility (time to administer, time to score, cost of using the measures, technological or instruction support needed, and staff training support needed).

The methodological quality of the included studies will be assessed against the COSMIN checklist, which was developed specifically for evaluating the methodological quality of studies on health-related outcome measurements [15,16]. A four-point scale, ‘excellent’, ‘good’, ‘fair’, and ‘poor’, will be used to score each measurement properties; study methodological quality will be rated for each measurement property evaluated within the study and determined by the lowest rating [16]. Two independent reviewers will perform the data extraction and the evaluation of the methodological quality of each selected study, and a third reviewer will resolve any disagreement.

### Data synthesis

Best evidence synthesis will be performed in this review as reported in other similar reviews [20-22]. This qualitative synthesis will determine the overall quality and acceptability of each identified instrument. This synthesis will be based on the following criterion: 1) the number of studies in which the measurement or practical properties of the instrument is assessed, 2) the homogeneity and methodological quality of these studies, 3) the results of each measurement/practical property per measure, and 4) the consistency of the results. The overall rating for outcome measures properties will be rated as ‘positive’, ‘indeterminate’, or ‘negative’ as reported by Terwee *et al*. (see Table 1) [23]. This will accompany the level of evidence (strong, moderate, limited, conflicting, unknown) as suggested by the Cochrane Back Review Group (see Table 2) [24,25]. The synthesis will produce a list of measures that are suitable for assessing upper limb function for neck pain patients and show the overall quality, acceptability, and feasibility of each of those measures.

**Table 1:** **Quality criteria for measurement properties** [23]

**Property**	**Rating** ^**a**^	**Quality criteria**
*Reliability*		
Internal consistency	+	Cronbach’s alpha(s) ≥ 0.70
	?	Cronbach’s alpha not determined or dimensionality unknown
	-	Cronbach’s alpha(s) < 0.70
Reliability	+	ICC/weighted Kappa ≥ 0.70 OR Pearson’s *r* ≥ 0.80
	?	Neither ICC/weighted Kappa, nor Pearson’s *r* determined
	-	ICC/weighted Kappa < 0.70 OR Pearson’s *r* < 0.80
Measurement error	+	MIC > SDC OR MIC outside the LOA
	?	MIC not defined
	-	MIC ≤ SDC OR MIC equals or inside LOA
*Validity*		
Content validity	+	All items are considered to be relevant for the construct to be measured, for the target population, and for the purpose of the measurement AND the questionnaire is considered to be comprehensive
	?	Not enough information available
	-	Not all items are considered to be relevant for the construct to be measured, for the target population, and for the purpose of the measurement OR the questionnaire is considered not to be comprehensive
Construct validity		
Structural validity	+	Factors should explain at least 50% of the variance
	?	Explained variance not mentioned
	-	Factors explain < 50% of the variance
Hypothesis testing	+	Correlations with instruments measuring the same construct ≥ 0.50 OR at least 75% of the results are in accordance with the hypotheses AND correlations with related constructs are higher than with unrelated constructs
	?	Solely correlations determined with unrelated constructs
	-	Correlations with instruments measuring the same construct < 0.50 OR < 75% of the results are in accordance with the hypotheses OR correlations with related constructs are lower than with unrelated constructs
Cross-cultural validity	+	No differences in factor structure OR no important DIF between language versions
	?	Multiple group factor analysis not applied AND DIF not assessed
	-	Differences in factor structure OR important DIF between language versions
Criterion validity	+	Convincing arguments that gold standard is ‘gold’ AND correlation with gold standard ≥ 0.70
	?	No convincing arguments that gold standard is ‘gold’
	-	Correlation with gold standard < 0.70
*Responsiveness*		
Responsiveness	+	Correlation with changes on instruments measuring the same construct ≥ 0.50 OR at least 75% of the results are in accordance with the hypotheses OR AUC ≥ 0.70 AND correlations with changes in related constructs are higher than with unrelated constructs
	?	Solely correlations determined with unrelated constructs
	-	Correlations with changes on instruments measuring the same construct < 0.50 OR < 75% of the results are in accordance with the hypotheses OR AUC < 0.70 OR correlations with changes in related constructs are lower than with unrelated constructs

MIC = minimal important change, SDC = smallest detectable change, LOA = limits of agreement, ICC = intraclass correlation coefficient, DIF = differential item functioning, AUC = area under the curve. ^a^ + = positive rating, ? = indeterminate rating, - = negative rating.

**Table 2:** **Levels of evidence for the overall quality of the measurement property** [24,25]

**Level**	**Rating** ^**a**^	**Criteria**
Strong	+++ or ---	Consistent findings in multiple studies of good methodological quality OR in one study of excellent methodological quality
Moderate		Consistent findings in multiple studies of fair methodological quality OR in one study of good methodological quality
Limited	+ or -	One study of fair methodological quality
Conflicting	+/−	Conflicting findings
Unknown	?	Only studies of poor methodological quality

^a^ + = positive result, - = negative result.

### Reporting

For the purpose of methodological rigour, the results of this study will be reported in accordance with the PRISMA guidelines [17]. This will report information with regard to the following aspects: 1) the results of the literature search (search strategies) and the inclusion of studies (presented in a flow chart), 2) the methodological quality of each study per measurement property, 3) the characteristics of identified outcome measure instruments, 4) the characteristics of included studies, 5) the quality of the measurement properties per instrument, and 6) conclusion about the best suitable outcome instrument for measuring upper limb function in patients with neck pain.

## Discussion

In clinical practice, the availability of valid and reliable upper limb measures of upper limb disability will support the recommended assessment and management of patients with neck disorders [5,11]. This systematic review will identify and critically examine the quality of all available measures that can be used to assess upper limb function in neck pain patients and identify the best available measure that is suitable and appropriate for use in clinical practice and research. Such a measure, which can accurately examine upper limb capacity and monitor the progress of patients during the rehabilitation programme, will enable clinicians to deliver safe, effective, and efficient treatment for patients with neck disorders. In addition, the availability of a valid and reliable measure will enable further robust clinical research to investigate the relationship between neck pain and upper limb disability. This may suggest new strategies to improve or prevent upper limb disability in neck pain patients. The findings of this review may also reveal gaps in research and suggest new research directions to support the further development of measure of upper limb function for patients with neck pain.

## Abbreviations

AMED: Allied and Complementary Medicine Database
CINAHL: Cumulative Index to Nursing and Allied Health Literature
COSMIN: COnsensus-based Standards for selection of health Measurement INstrument
MIC: minimal important change
PRISMA: Preferred Reporting Items for Systematic Review and Meta-Analysis
SDC: smallest detectable changes
